# Implications of the lack of desiccation tolerance in recalcitrant seeds

**DOI:** 10.3389/fpls.2013.00478

**Published:** 2013-11-22

**Authors:** Patricia Berjak, Norman W. Pammenter

**Affiliations:** Plant Germplasm Conservation Research, School of Life Sciences, University of KwaZulu-Natal (Westville Campus)Durban, South Africa

**Keywords:** cryopreservation, dehydration, desiccation damage, desiccation sensitivity, ice crystallization, recalcitrant seeds, ROS, vitrification

## Abstract

A suite of interacting processes and mechanisms enables tolerance of desiccation and storage (conservation) of orthodox seeds in the dry state. While this is a long-term option under optimized conditions, dry orthodox seeds are not immortal, with life spans having been characterized as short, intermediate and long. Factors facilitating desiccation tolerance are metabolic “switch-off” and intracellular dedifferentiation. Recalcitrant seeds lack these mechanisms, contributing significantly to their desiccation sensitivity. Consequently, recalcitrant seeds, which are shed at high water contents, can be stored only in the short-term, under conditions not allowing dehydration. The periods of such hydrated storage are constrained by germination that occurs without the need for extraneous water, and the proliferation of seed-associated fungi. Cryopreservation is viewed as the only option for long-term conservation of the germplasm of recalcitrant-seeded species. This is not easily achieved, as each of the necessary procedures imposes oxidative damage. Intact recalcitrant seeds cannot be cryopreserved, the common practice being to use excised embryos or embryonic axes as explants. Dehydration is a necessary procedure prior to exposure to cryogenic temperatures, but this is associated with metabolism-linked injury mediated by uncontrolled reactive oxygen species generation and failing anti-oxidant systems. While the extent to which this occurs can be curtailed by maximizing drying rate (flash drying) it cannot be completely obviated. Explant cooling for, and rewarming after, cryostorage must necessarily be rapid, to avoid ice crystallization. The ramifications of desiccation sensitivity are discussed, as are problems involved in cryostorage, particularly those accompanying dehydration and damage consequent upon ice crystallization. While desiccation sensitivity is a “fact” of seed recalcitrance, resolutions of the difficulties involved germplasm conservation are possible as discussed.

## INTRODUCTION

The most convenient means of *ex situ* conservation of plant germplasm is by storage of seeds under conditions which maximize their post-harvest life spans. For desiccation-tolerant (orthodox) seeds it is recommended that this be achieved by their storage in the dry state in equilibrium with low relative humidity (RH) – around 15% – and sub-zero temperatures, generally -18° C, which are the norms in most seed banks (FAO, 2013). However, there is a spectrum of species which produce seeds that are not desiccation-tolerant (recalcitrant-seeded species) and those which producing few or no viable seeds, for which the collective term, “exceptional species,” is being promoted (Pence, 2011) and this grouping could be expanded to include seeds having very short life spans in the dry state. While the genetic resources of poor- or non-seeding species have traditionally been multiplied and conserved as *in vitro* cultures, there is now a drive for long-term cryopreservation of explants (usually shoot apices/meristems) and germplasm derived from recalcitrant seeds (Keller et al., 2010; Reed et al., 2011). Cryopreservation generally entails storage of living material in liquid nitrogen (LN) at -196° C or in the vapor phase over LN in the range -160 to 140° C. While the focus of this review is on the implications of desiccation sensitivity of recalcitrant seeds in the context of germplasm conservation, the characteristics of desiccation-tolerant orthodox seeds which enable traditional banking are briefly considered first.

## WHAT UNDERLIES SEED SURVIVAL IN THE DESICCATED STATE?

Understanding of the basis of seed desiccation sensitivity starts with knowledge of processes and mechanisms involved in the acquisition and maintenance of desiccation tolerance in orthodox seeds, and querying whether or not these occur in recalcitrant types. Although there are some differences, there is considerable commonality in events conferring desiccation tolerance across a broad spectrum of life forms (Berjak, 2006) and particular parallels between seeds and vegetative tissues of resurrection plants have emerged in which re-activation of latent seed-specific genetic elements is apparent (Farrant and Moore, 2011).

From fertilization onward, angiosperm seeds progress through three developmental stages, *viz*. histodifferentiation characterized by intensive metabolic activity and marked desiccation sensitivity, accumulation of reserves, and maturation – which, in orthodox seeds is characterized by a plateau in dry mass and usually (but not invariably) the significant decline in water content to very low levels. Those orthodox seeds which do not dry substantially during maturation, e.g., tomato (Demir and Ellis, 1992), nevertheless withstand desiccation after removal from the fruit. The acquisition of desiccation tolerance occurs during reserve accumulation phase so that, at the start of maturation drying, the seed tissues are predisposed to withstand the stresses imposed during dehydration (e.g., Kermode and Finch-Savage, 2002). In developing seeds of desiccation-sensitive exceptional species, it is the inability – or perhaps only the very limited ability – to express or entrain the processes and mechanisms conferring desiccation tolerance, which preclude the dramatic decline in water content during the final phase of pre-shedding development.

The major features which become apparent in embryo cells, the underlying mechanisms and processes involved and their interactions in developing seeds upon acquisition of desiccation tolerance, have been sequentially extensively reviewed (Vertucci and Farrant, 1995; Pammenter and Berjak, 1999; Kermode and Finch-Savage, 2002; Berjak et al., 2007; Berjak and Pammenter, 2008). Consequently, to provide the context for comparison of the desiccation-sensitive seed situation, these are briefly described, but not detailed here.

Intracellular physical characteristics which become modified with the acquisition of desiccation tolerance include deposition of insoluble proteins within vacuoles, thus providing mechanical resilience against cell collapse. Accumulated starch and lipid reserves provide further volume buffering capacity. Condensation of the chromatin coinciding with cessation of replication and transcription, and orderly dismantling of the cytoskeleton are further features noted to be associated with the acquisition of desiccation tolerance.

Late embryogenesis abundant proteins, the LEAs, are very important feature in the acquisition of desiccation tolerance. The LEAs, of which several groups have been identified based on particular peptide motifs, are small hydrophilic, heat-stable molecules which are unstructured in solution. While their exact modes of action in providing protection during desiccation is still conjectural, the considerable amount of focused proteomics work over the past few years has unequivocally linked certain LEAs with desiccation tolerance in orthodox seeds (see Delahaie et al., 2013 and references therein).

Metabolic “switch off,” dedifferentiation of organelles and reduction of the elements of the endomembrane system interact, thus limiting unregulated metabolism and the membranous targets of consequent generation of free radicals/reactive oxygen species (ROS). The latter would be a particular hazard as water contents and potentials decline through “intermediate” ranges. Over-arching all these phenomena is the presence and activity of anti-oxidants appropriate to the various declining hydration levels experienced by the cells during dehydration.

High concentrations of sucrose with certain raffinose series oligosaccharides accumulated in orthodox seeds, together with LEAs, are major constituents contributing to the intracellular vitrified (glassy) state in seeds once the dry-mass-specific water concentration is ≤0.3 g g^-^^1^ dry mass. It is important to note though, that many other cytomatrical component molecules must be incorporated in the glassy matrix. Vitrification imposes a highly viscous intracellular situation, so limiting residual reactivity and the migration of free radicals/ROS. Maintenance of intracellular vitrification is intrinsic to seed survival in the desiccated condition but, because it is metastable, the glassy state does not confer immortality even under very cold storage conditions.

## STORAGE OF DESICCATION-TOLERANT SEEDS

Seed/gene banks generally store orthodox seeds in the long term using conventional or walk-in freezers at -18° C, although accessions are also maintained in LN (Pritchard and Nadarajan, 2008). However, prior to storage it is essential that seeds of uncharacterized species are screened to ensure that they are of the desiccation-tolerant type. Before banking any accession, good seed quality must have been established, which should include checking for associated fungal contamination. In conventional banking practice, seed samples are then cleaned, after which they are equilibrated at 10–25% RH and 5–20° C, depending on the species and finally sealed in air-tight containers which are then placed in the freezer for long-term storage (FAO, 2013). It is the absence of any freezable water (see Ramifications of the cooling process) which enables desiccated orthodox seeds to be stored at sub-zero temperatures. Medium-term storage is generally under refrigerated conditions, but for storage in the short term, cool, stable ambient conditions are considered to be adequate (FAO, 2013).

Orthodox seed longevity in storage has long been known to increase with decreasing storage temperature and seed water (moisture) content and *vice versa* (Harrington, 1972) and, in fact, is predictable under sets of defined parameters (Ellis and Roberts, 1980). Despite their desiccation tolerance, it has become apparent that there is a water content limit below which no additional improvement in orthodox seed storage life span will result (Ellis and Hong, 1996). It has also been contended that dehydration below such critical levels may actually be detrimental (Walters, 1998), although there is some controversy in this regard (Pérez-Garcia et al., 2009). Critical water contents, which vary with seed type/composition are best ascertained using water sorption isotherms which indicate the amount of water in the seed at different equilibrium RHs (eRH) that are temperature dependent.

There is, however, considerable variability in longevity of high-quality, mature orthodox seeds across species stored under ambient conditions (Nagel and Börner, 2010) as well as those maintained in the cold at both 5 and -18° C (Walters et al., 2005). Based on *P*_50_ values (i.e., the storage time after which viability had declined to 50%) of some 42,000 accessions representing 276 species, Walters et al. (2005) were able to categorize the longevity of the seeds as inherently being short (<25 y), medium (30–70 y) or long (80–500 y). Those authors also found that there was no correlation among the *P*_50_ values and content of sucrose or oligosaccharides. Studies such as those indicate that although orthodox seeds share the property of desiccation tolerance, there are significant intrinsic differences which determine the period for which they will retain viability under similar water content determined by eRH and temperature conditions.

The study of Walters et al. (2005) showed that, while seeds of particular families shared the trait of being short- or long-lived, there was also commonality based on provenance. Seeds originating from temperate, moist environments generally had short storage life spans while those from warm, arid regions were long-lived. Those observations have been supported by work on 195 species by Probert et al. (2009), who also found that endospermous seeds tended to have shorter life spans than ex-endospermous types. However, whatever the inherent life span of orthodox seeds of individual species, it is essential that the conditions of storage [including packaging (Gómez-Campo, 2006; Walters, 2007)] are such as to maximize longevity.

Although diametrically different from orthodox seeds in terms of their physiology and responses, desiccation-sensitive recalcitrant seeds demand equal – or even greater – attention to fine detail in terms of short-term storage and long-term germplasm conservation. If these endeavors are not to be merely empirical, they must be based on an appreciation of the underlying nature of seed recalcitrance.

## TRAITS OF RECALCITRANT SEEDS

### BACKGROUND

Recalcitrant seeds (*sensu* Roberts, 1973) were so-named on the basis of their post-harvest viability loss in response to dehydration [although the descriptor is also used in other contexts, e.g., as cryostorage recalcitrance, for explants that are seemingly unable to recover in culture after exposure to cryogenic temperatures (Benson, 2008)]. Among their traits, recalcitrant seeds are very variable both within and across species as well as inter- and intra-seasonally. For example, seeds of *Camellia sinensis* harvested from the same tree population had mean water contents as different as 4.4 and 2.0 g g^-^^1^ in consecutive years (Berjak and Pammenter, 2008). Xia et al. (2012), investigating ranges of species of *Quercus* and *Cyclobalanopsis* seeds, reported wide differences in the rates at which the seeds dried when dehydrated from 1.0 g g^-^^1^ under identical conditions. Recalcitrant seeds either lack, or do not express, the various processes and mechanisms typifying the acquisition of desiccation tolerance as occurs in orthodox seeds. As an example, recent work on recalcitrant seeds of *Castanospermum australe*, has revealed the absence of LEAs which have been shown to be critical for desiccation tolerance (Delahaie et al., 2013).

Because they are shed at a range of species-related high water contents and will not tolerate dehydration to levels appropriate for low RH/sub-zero temperature storage, recalcitrant seeds cannot be conserved using standard genebank approaches. Short-/medium-term storage is possible, but the conditions must preclude water loss. However, as recalcitrant seeds are not only metabolically active, but will germinate at the water contents typifying shedding, such hydrated storage is strictly a short-term option (Berjak and Pammenter, 2008). Additionally, proliferation of seed-associated fungi under hydrated storage conditions is an enduring problem, especially for seeds of tropical/sub-tropical provenance, which almost invariably harbor fungal inoculum internally (Sutherland et al., 2002). While short-term storage of recalcitrant seeds under the best conditions possible is necessary (see below), currently the only feasible option for long-term storage of the germplasm is by cryopreservation (Engelmann, 2011), which entails complex processing and *in vitro* recovery presently requiring to be refined on an individual species basis.

### IMPLICATIONS OF DESICCATION SENSITIVITY OF RECALCITRANT SEEDS FOR SHORT- TO MEDIUM-TERM STORAGE

Lack of the ability for metabolic switch-off as occurs in orthodox seeds, is one of the possible basic reasons that recalcitrant seeds are desiccation sensitive. Metabolism progresses without any obvious punctuation from development into germination, the onset of which strictly curtails the period for which the seeds can be stored. Nevertheless, hydrated storage under saturated RH conditions, is necessary to maintain viability in the short- to medium-term. This is undertaken in the short- to medium-term (the length of which is related to the degree of species-specific embryo development when the seeds are shed) for: propagation soon after harvest; for the use of seeds in experiments to ascertain their characteristics and post-harvest responses – which differ not only among species, but frequently within species inter- and intra-seasonally; and as stocks to be used when cryostorage protocols are being developed for excised embryos or embryonic axes. However, the seeds will sooner or later germinate in storage, and because those of many tropical/sub-tropical species are chilling-sensitive (Berjak and Pammenter, 2008), preliminary trials must be undertaken to ascertain the lowest temperature which will limit metabolic progress while having no adverse effects on viability. In this context also, dehydration to “sub-lethal” water contents – originally suggested as a means to slow or obviate germination in storage (King and Roberts, 1980) – actually enhances the rate of germination (e.g., Fu et al., 1994; Tompsett and Pritchard, 1998).

As the processes involved in germination progress, recalcitrant seeds become increasingly desiccation sensitive (Farrant et al., 1989; Pammenter and Berjak, 1999): the finding that both germination rate and desiccation sensitivity were enhanced when the seeds of several species were subjected to mild dehydration (Drew et al., 2000; Eggers et al., 2007) is in agreement with those earlier observations. Enhancement of germination rate is suggested to be a natural response to mild water stress which promotes the rate of seedling establishment: once rooted, a seedling would have access to soil water not available to the seed.

Hydrated storage conditions which generally involve benign temperatures, also encourage fungal proliferation, which is perhaps the most serious problem – not only for intact seeds, but also when embryos/axes which need to be cultured *in vitro*, are used as explants for cryopreservation. This necessitates the development of effective means not only for surface decontamination of the seeds, but also of the explants. As decontaminants, e.g., sodium hypochlorite (NaOCl), are harsh oxidizing agents, development of non-injurious procedures is frequently challenging. Although NaOCl as a 1% (*m/v*) solution (a 3:1 dilution of commercial bleach) is among the most commonly used decontaminants in plant tissue culture, we have found sodium dichloroisocyanurate (NaDCC) used as a 0.03–0.05% *w/v* solution to be more effective and less phytotoxic, as reviewed by Barnicoat et al. (2011). Nevertheless, the seeds which should have been harvested directly from the mother plants to obviate further contamination by soil-associated micro-organisms, need to be in the best possible condition so as to avoid internal contaminants initially on embryo surfaces, becoming established to the point of being impossible to eliminate.

### DRYING AND DRYING RATE

As discussed above in the context of the acquisition of desiccation tolerance in orthodox seeds, there is a reduction in vacuolar volume, often achieved by filling these compartments with insoluble material. Vacuoles are prominent in cells of the embryonic axes of mature recalcitrant seeds, and seemingly, the more desiccation sensitive the seed the greater is the degree of vacuolation (Farrant et al., 1997). Even in axes of *Aesculus hippocastanum* which might be best described as being “moderately recalcitrant” from the data of Farrant et al. (1997), ultrastructural and biochemical evidence attests to vacuolar activity during cold stratification with initiation of germinative growth being accompanied by vacuole enlargement and heightened invertase activity (Obroucheva et al., 2012). In view of mechanical damage associated with volume reduction when water is lost (Iljin, 1957) one could suggest that vacuolar collapse occurring in cells of tissues not “primed” by reducing/filling these compartments, is an important factor in the damage accrued by recalcitrant seeds, embryos and embryonic axes.

Although it may seem paradoxical to discuss the effects of drying rate when addressing the implications of the lack of desiccation tolerance of recalcitrant seeds, observations that more rapid dehydration facilitates survival to lower water contents than does slow drying (Berjak et al., 1984; Farrant et al., 1985; Pritchard, 1991) added significantly to our understanding of aspects of recalcitrance. In particular also, this finding afforded a breakthrough in the technology involved in preparing embryos/embryonic axes for cryopreservation (Normah et al., 1986; Berjak et al., 1990) which was developed during the 1990s as flash drying (Pammenter et al., 2002). These findings emphasize the futility of mention of “critical” water content levels at which viability is adversely affected or lost, without these being qualified in terms of the drying rate (Pammenter et al., 1998).

The explanation for the effects of rapid drying in facilitating survival of a greater degree of dehydration resides in the fact that recalcitrant seeds, embryos/embryonic axes are metabolically active. As water is lost, aqueous-based metabolism continues, but becomes unbalanced, so that ROS are generated while anti-oxidant systems fail (Côme and Corbineau, 1996; Varghese et al., 2011). This is termed metabolism-linked damage (Walters et al., 2001). If dehydration is slow, the specimens spend extended times at “intermediate” water contents where aqueous-based metabolism-linked damage accumulates, with the consequence that viability is lost at relatively high water contents – e.g., around 1.0 g g^-^^1^ for *Ekebergia capensis* axes (Pammenter et al., 1998). Under conditions of rapid drying, the time during which metabolism-linked damage occurs is curtailed, so that considerably lower water contents commensurate with viability retention are achieved. However, it must be appreciated that recalcitrant seeds, embryos and embryonic axes are not inherently desiccation tolerant, nor does rapid dehydration achieve such tolerance. Even when dried rapidly, recalcitrant material will not survive for longer than a day or two under ambient or above-zero refrigerated conditions (Walters et al., 2001). However, what is achieved by dehydrating excised embryos or embryonic axes rapidly, is their immediate availability as explants for cryopreservation.

No matter how rapidly recalcitrant material is able to be dehydrated, there is a water content limit below which viability will be lost, which is about an order of magnitude higher than that survived by orthodox seeds. This is may be coincident with, but generally is slightly higher than, the level (≤0.35 g g^-^^1^) at which all the remaining water is non-freezable (Pammenter et al., 1991, 1993; Pritchard, 1991; Finch-Savage, 1992; Berjak et al., 1993; Pritchard and Manger, 1998).

There is one *caveat* to be borne in mind when considering values for water contents, and that is the possibility of uneven drying which has been shown to occur between different organs within whole recalcitrant seeds, e.g., evergreen Asian oaks (Xia et al., 2012). For embryos or axes, water contents are generally measured gravimetrically but, during flash drying of e.g., embryonic axes, water is initially likely to be lost from the apoplast and outer cell layers before being removed from deeper-lying tissues. Water contents of individual axes cannot reveal differences in status between, e.g., cortical and meristematic tissues, and it may be that the latter remain more hydrated, and therefore less prone to desiccation damage *sensu stricto*. This is an aspect currently receiving attention in our laboratory.

### DEHYDRATION DAMAGE IN THE CONTEXT OF EXPLANT CRYOPRESERVATION

*In vitro* culture and seedling slow-growth notwithstanding, the only means of effective long-term conservation of the germplasm of recalcitrant-seeded species is by cryopreservation (Engelmann, 2011). Recalcitrant seeds of most species are large, and inevitably, are hydrated and metabolically active. In general, their size (among other characteristics) precludes the rapid drying that is an essential prerequisite for viability retention to sufficiently low water contents prior to exposure to cryogenic temperatures (Berjak and Pammenter, 2008). Large recalcitrant seeds also cannot be sufficiently rapidly cooled to LN temperature, which further precludes their cryopreservation. Consequently, embryos or more commonly, embryonic axes, both of which are representative of the genetic diversity of the seeds, are the explants of choice for cryopreservation. This entails excision of the embryos/axes from the seeds which imposes the first of the oxidative stresses inflicted during processing for, and retrieval from, cryostorage. [However, as the subject of this review focuses on the implications of the lack of desiccation tolerance, excision-related oxidative injury as first suggested by Goveia et al. (2004) and studied in detail by Roach et al. (2008), Whitaker et al. (2010), Berjak et al. (2011), Naidoo et al. (2011), and Varghese et al. (2011), will not presently be considered further.

There are two other major causes of damage associated with cryopreservation, viz. dehydration, and ice crystallization, and, prior to cryogen exposure, explants must be partially dehydrated to water contents generally curtailing (although ideally precluding) ice formation in the tissues. For embryos/embryonic axes, this is usually achieved by flash drying (Pammenter et al., 2002). However, recalcitrant embryos/axes cannot survive the loss of non-freezable (structure-associated) water, and are enduringly metabolically active; both characteristics are factors causing problems for successful cryopreservation. Below the limit at which all intracellular water is non-freezable (≤0.35 g g^-^^1^), further dehydration will withdraw it from intracellular structures and macromolecules: this category or type of damage is termed desiccation damage *sensu stricto* (Walters et al., 2001). Perturbation of structure-associated water is lethal for recalcitrant material but this is obviously not the case for orthodox seeds. While the reasons for this have not been established, the explanation must reside in one or more of the protective mechanisms entrained when desiccation tolerance is acquired in orthodox seeds.

While metabolic activity is reduced it is not obviated by flash drying, and the potential remains for damaging levels of ROS production and/or failure of the anti-oxidant system (Varghese et al., 2011). ROS are now widely recognized as signal transducers in plants, and extracellularly produced superoxide (O•2−), hydrogen peroxide (H_2_O_2_), and the hydroxyl radical (^•^OH) have been implicated in events during germination and seedling establishment of orthodox seeds (Müller et al., 2009; Kranner et al., 2010). However, when normally produced in vegetative plant tissues and germinating seeds, the ROS are under strict control, and there must be a fine balance between their production and scavenging (Halliwell, 2006; Van Breusegem et al., 2008). While parallels must exist in recalcitrant seeds, no base-line data for axes that are not obscured by the phenomenon of the excision-related ROS burst, are available. However, dehydration of desiccation sensitive recalcitrant seed tissues is accompanied by an imbalance between ROS production and anti-oxidant capacity, with particular attention having been paid to O•2−, ^•^OH, H_2_O_2_, and singlet oxygen (^1^O_2_) (C^o^me and Corbineau, 1996; Varghese and Naithani, 2002; Bailly, 2004; Kranner and Birtić, 2005; Pukacka and Ratajczak, 2006; Berjak and Pammenter, 2008; Varghese et al., 2011).

The data for ROS production and anti-oxidant activity in recalcitrant seed tissues in response to dehydration are inconsistent across species. However, this is not really surprising in view of the marked variability among recalcitrant seeds in terms of water content at shedding, rate at which water is lost and responses to drying (Berjak and Pammenter, 2008). For example there is a lessening of the degree of sensitivity when seeds develop under warm temperature conditions that favor extended progression of the phase of reserve accumulation (Daws et al., 2004, 2006; Daws and Jensen, 2011). This phenomenon, which has been described as the outcome of developmental heat sum by those authors, has a marked influence on the responses of recalcitrant seeds to dehydration. Furthermore, there are considerable differences in the modes, rates and duration of drying employed by various investigators.

Results showings similar trends, however, have been reported by a number of investigators. For example, increased levels of ROS in embryonic axes were reported by Hendry (1993) who dried *Quercus robur* seeds after pericarp removal in an air-flow at 30% RH and 20° C and associated viability loss with failing anti-oxidant activity. Pukacka and Ratajczak (2006), who dried silver maple (*Acer saccharinum*) seeds for 14 days under conditions of 35–40% RH and 20–23° C, reported that loss of viability was accompanied by increases in both O•2− and H_2_O_2_, while after a transitory increase, the levels and redox status of ascorbic acid and the reduced form of glutathione (GHS) decreased rapidly. The ratio of reduced to oxidized glutathione (GSSG) has been reported generally to decline as a stress response in desiccation-sensitive seed tissues, indicating an increasingly oxidized state (Pukacka and Ratajczak, 2006; Tommasi et al., 2006; Varghese et al., 2011). Ratajczak et al. (2013) reported differences between orthodox *Acer platanoides* and recalcitrant *Acer pseudoplatanus* seeds in the mitochondrial peroxiredoxin IIF transcript and protein levels throughout desiccation from a mean water content of 1.0 g g^-^^1^. Those authors, who also demonstrated differences between desiccation-tolerant *Acer platanoides* and desiccation-sensitive *Acer pseudoplatanus* in the level of post-translational modification of the mitochondrial peroxiredoxin suggested that mitochondrial redox homeostasis might be one of the defining features of viability maintenance upon desiccation.

Increased ROS accumulation was noted by Cheng and Song (2008) to accompany dehydration and viability loss of recalcitrant *Antiaris toxiacaria* axes over 6 days at 45% RH and 15° C. Those authors recorded a dehydration-associated initial increase, followed by declining activity of a spectrum of enzymic anti-oxidants, as noted for non-enzymic anti-oxidants (Pukacka and Ratajczak, 2006). In contrast, when axes of *Antiaris toxiacaria* were rapidly dried, there was no significant correlation between viability loss and accumulation of ROS or lipid peroxidation (Xin et al., 2010). In agreement with those observations, Varghese et al. (2011) reported that flash drying of axes of *Trichilia dregeana* had no consistent effect on either ROS generation or anti-oxidant activity. However, on slow seed drying over 60 days, whilst there was no relationship between viability and ROS accumulation in the axes, there was a linear decline in anti-oxidant activity as viability was lost.

It is important that the means are developed to maximize protection against oxidative damage at all stages of the cryoprocess, and upon explant retrieval from cryostorage, and some progress has been made. In this regard, Bai et al. (2011) have reported that nitric oxide enhances survival of *A. toxicaria* axes and Pukacka et al. (2011) have found that provision of selenium protects *Acer saccharinum* axes subjected to desiccation. The use of cathodic water (produced by electrolysis of a dilute solution of calcium- and magnesium chloride) that has strong reducing properties, is promising in terms of ROS quenching (Berjak et al., 2011).

### PARTIAL DEHYDRATION USING CRYOPROTECTANTS

Recalcitrant embryo/axis dehydration is a necessary procedure preceding cryogen exposure. When working with shoot apices/meristems, it is common practice to expose them to cryoprotectants. These are chemical substances that concentrate cell contents to the extent that homogeneous intracellular solidification without the formation of crystalline ice (i.e., the glassy/vitrified state) should occur upon cooling to cryogenic temperatures (Engelmann, 2011). Most simply stated, cryoprotectants such as glycerol and dimethyl sulphoxide (DMSO) penetrate cells, so increasing solute concentration (among other effects), while non-penetrating substances such as sucrose and polyethylene glycol effect dehydration osmotically. Generally, cryoprotectant solutions are comprised of both penetrating and non-penetrating components (Panis and Lambardi, 2005). While partial cryoprotectant-mediated dehydration prior to flash drying has proved variably successful for embryos of some herbaceous amaryllid species (Sershen et al., 2007), seed-derived explants of woody tropical/sub-tropical species are generally adversely affected (Engelmann, 2011; Normah et al., 2011). Presently, therefore, rapid physical dehydration remains the best option.

### RAMIFICATIONS OF THE COOLING PROCESS

While the ideal hydration status of recalcitrant explants prior to cryogen exposure would be that they contain only non-freezable water, in practice this presently is not attainable. The variation in axis water content at the start continues to be reflected after drying, indicating that when a mean water content of (say) 0.35 g g^-^^1^ is obtained, some axes would have been over-dried, while others could be at considerably higher water contents. As discussed earlier, recalcitrant material will not withstand perturbation of the structure-associated, non-freezable water. Considering that the objective is to remove as little water as is necessary to ensure explant survival of cryogenic cooling, in practice inevitably some solution water which is freezable will remain in the cells.

It has long been held that formation of large ice crystals intracellularly will be lethal (Meryman, 1968) which underlies development of treatments to achieve intracellular vitrification. However, cryoprotectants generally have adverse effects on recalcitrant axes of woody tropical/sub-tropical species directly, or after further drying, or upon cooling. Furthermore, such explants do not generally seem to survive exposure to plant vitrification solutions (e.g., PVS2) formulated by Sakai et al. (1990). This solution of cryoprotectants, which is 7.8M (Sakai and Engelmann, 2007) has been extensively and successfully used in cryopreservation protocols for apices and meristems (Engelmann, 2011) and the basis for its lethal effects on embryonic axes needs resolution.

This effectively leaves only flash drying as the means to bring about suitable intracellular concentration, coupled with manipulation of the cooling rate. While a uniformly vitrified state is unlikely across the complex tissues of embryos/axes, optimizing cooling rate to limit ice crystallization *per se*, or to restrict crystal size to being so small as to be innocuous, are obvious aims. In this context, work on axes of *Acer saccharinum* showed that drying did reduce ice crystal size to levels undetectable with the transmission electron microscope (Wesley-Smith et al., 2013). However, those authors also indicated that radicle cells of partially hydrated axes would survive considerable ice formation when crystal size and density was restricted to ~2–4 μm and 10–20 crystals per μm^-^^2^, respectively. Concomitant with survival, fewer – or no – crystals were detectable within the lumen of the endoplasmic reticulum, mitochondria and plastids (Wesley-Smith et al., 2013). Interestingly, however, shoot meristems were more susceptible to damage than were root meristems.

### THE IMPORTANCE OF COOLING AND WARMING RATES

The ideal for cryopreservation is to induce vitrification. The glassy state is one of extremely high viscocity (it is effectively a solid in nature), such that molecular mobility is extremely low. The vitrified state will prevent ice crystal formation and growth to sizes sufficiently large to cause mechanical damage (although indications are that the production of small ice crystals may not be lethal – see above). The low molecular ability in the glassy matrix will also impose a stasis on the generation of metabolically derived ROS.

As pure water is cooled the diffusional mobility of the individual water molecules is reduced, increasing the likelihood of an “embryo” ice crystal being sufficiently stable to grow: this is a phenomenon known as homogeneous nucleation which, in pure water, will occur at -40° C. Irregularities in the water (as afforded by intracellular surfaces) provide nucleation sites on which ice crystals can form and grow: this is the process of heterogeneous nucleation (also referred to as “seeding”) and will occur at temperatures higher than -40° C. It is likely that ice crystal formation in biological tissues is via heterogeneous nucleation and fully hydrated cells freezing will be initiated at about -2° C.

Although homogeneous nucleation will occur at -40° C, at temperatures below -80° C, because molecular mobility has been further reduced, ice crystal is arrested (Moor, 1973). Thus there is a temperature range from just below 0 to about -80° C where ice crystal formation and growth is possible: the longer the tissue resides in this temperature range the more likely is ice crystal damage. It is for this reason that most cryopreservation protocols for complex multicellular tissues include very rapid cooling (of the order of hundreds of degrees per second), rates that require very small pieces of tissue. Similarly, on warming of the tissue after cryogen exposure, the vitrified state of water can “relax” and convert to the crystalline state, generating physical damage associated with ice crystals. Also in this temperature range, there is the danger of small crystals annealing to form larger masses. As with cooling, the longer the tissue is exposed to this warming process, the more the likelihood of ice crystal formation, and so rapid warming is as important as rapid cooling.

## CONCLUDING COMMENTS

Desiccation tolerance, although not common, occurs across a range of life forms and there appear to be some commonalities in the mechanisms proposed to confer tolerance. There is one group in which tolerance is common – the seeds of spermatophytes. The work on seeds, particularly storage of seeds for germplasm conservation, has demonstrated that desiccation tolerance is not just desiccation tolerance; there is a time component to tolerance in the dry state.

Sensitivity to dehydration implies that something that confers tolerance is missing from the sensitive tissue, but this has not yet been identified. It is much more likely, of course, that it is a number of putative mechanisms that are missing or fail, rather than one individual process. There are at least three levels at which damage occurs on drying of desiccation-sensitive tissue: mechanical damage consequent on reduction in volume; metabolism-induced ROS production on removal of water at intermediate hydration levels where aqueous-based processes can occur; and desiccation damage *sensu stricto* when water at low hydration levels and presumably bound to macromolecular structures, is removed. Tolerant tissue can obvious survive these potential stresses; sensitive tissue cannot.

At an applied level, desiccation sensitivity imposes constraints on long-term germplasm conservation by the inability for seed storage at low temperatures and water contents. Current approaches to resolving this problem are outlined. These involve reducing oxidative stress during drying and inducing vitrification by partial drying and rapid cooling (and subsequent warming) processes.

## Conflict of Interest Statement

The authors declare that the research was conducted in the absence of any commercial or financial relationships that could be construed as a potential conflict of interest.
