# Corrigendum: implications of the lack of desiccation tolerance in recalcitrant seeds

**DOI:** 10.3389/fpls.2014.00123

**Published:** 2014-04-03

**Authors:** Patricia Berjak, Norman W. Pammenter

**Affiliations:** Plant Germplasm Conservation Research, School of Life Sciences, University of KwaZulu-NatalDurban, South Africa

**Keywords:** cryopreservation, dehydration, desiccation damage, desiccation sensitivity, ice crystallization, recalcitrant

An error appears on p. 3, column 2, 4th line from end. The concentrations of NaDCC should appear as 0.30–0.50% (and *not* as 0.03 – 0.05%).

## Conflict of interest statement

The authors declare that the research was conducted in the absence of any commercial or financial relationships that could be construed as a potential conflict of interest.

